# Association of British Postal Medical Officers

**Published:** 1903-07-25

**Authors:** 


### ASSOCIATION OF BRITISH POSTAL MEDICAL OFFICERS.

DR. J. WALTON BROWNE, the President, took the chair at the annual dinner of the Association of British Postal Medical Officers at the Hôtel Métropole on Friday last, 17th inst.

The toast of the King having been duly honoured, Sir William Wills proposed the health of the Imperial Forces, which was responded to by Sir Henry Norbury, Director-General of the Medical Department of the Admiralty.

The Chairman, in proposing the health of the guest of the evening, the Right Hon. Austen Chamberlain, M.P., His Majesty's Postmaster-General, said they were proud to entertain the chief of that department to which they as medical officers were attached. He came of a good stock, and they hoped he would have as successful a career as his father, the Colonial Secretary. (Cheers.) He had had a careful training, and seemed determined to redress the grievances some of the officials of his department thought they were suffering from. He tendered their thanks to him for coming there that evening, and assured him it would always be their pleasure, as it was their duty, to assist him in any way in their power in the discharge of the responsibilities of his office.

Mr. Austen Chamberlain, on rising to respond, was greeted with loud cheers. He thanked the gathering sincerely for the cordial way they had honoured the toast and the chairman for the kindly terms in which he had proposed it. He accepted their assurance that they would always afford loyal support to the minister who occupied his position and to the heads of the department, and he could assure them that their services were not underrated by the chiefs of the service. He was conscious of how much they owed, and how much depended on their skill, their tact, and their judgment. No postmaster, and no post office was free from criticism, and could not hope to be. Every branch in turn was subjected to it, and even the medical officers attached to the department did not wholly escape. But he must congratulate them that they were masters in their own house, and he would be a very bold and rash Postmaster-General who would venture to differ from his medical officers on medical subjects; indeed, he sometimes wondered if the non-professional officials did not wish he would attach the same kind of professional sanctity, to their opinions that he was bound to admit in the case of the medical officers (laughter). The Post Office employed an enormous staff, the postal and telegraph servants exceeding 180,000. That association had the responsibility of keeping that great staff in perfectly good health so that their work could be efficiently carried on with that good temper which was the natural concomitant of good health and so important to the proper discharge of their duties. Whenever there was more than a very small number of employees attached to a post-office they endeavoured to secure the services of some medical gentleman to give medical attendance to the post office servants free of cost to themselves, and he would be glad if he could extend the benefits of those services to all the members of their widely-scattered staff and so meet the difficulty pointed out to him by the spokesman of a deputation that had waited on him, who urged that in the outlying districts where the wages were least the Post Office made the least provision. He recognised that the position of the medical officers was not always an easy one. He had to look to them that the very generous sick leave granted by the department was not abused to the detriment of the public service, and to rely on their tact, courage, and independence. It was because he found these qualities among them that he set so high a value on their services; and he hoped in the future to make greater and not less use of their services. He valued the good feeling shown there that night, and he was glad of that opportunity, the first of these annual gatherings since he became Postmaster General, of meeting them. He hoped their relations would continue to be as cordial and as satisfactory to both parties as hitherto. (Cheers.)

Mr. Edward Bond, M.P., proposed "The Colonies," which was responded to by Mr. Fuller, the Agent-General for Cape Colony, who said that the chief influences making for the maintenance of the bond between the Colonies and the Mother Country were family ties and the love of freedom. Their ideal must be to keep

Distinct as the branches,
Yet one as the tree;
Distinct as the waves,
Yet one as the sea.

Sir Henry Burdett proposed "The Houses of Parliament." They embodied, he said, the national sentiment and the national safety. He thought they might do a little more than they did to further national progress. If the younger peers would remember that the House of Lords still stood unequalled as a debating force they might get many social questions pressed forward. He believed that the House of Commons would render a great national service if they would attend more strictly to business. They were a business nation, and the great spending departments must be run on business lines. It was stated that the Government had in view the setting up of committees which should look closely into the affairs of the great spending departments. He hoped they would not fail, as was the case 20 years ago, because the Members considered it a very difficult subject, and so it was difficult to find men capable of serving on the committees. The nation ought not to sanction the appointment of a man to be head of the War Office or the Admiralty unless he was a man of business. If they insisted on having not only income and expenditure accounts, but capital accounts too for these two great departments, a saving of some six millions per annum might be effected. As a political economist he wanted to know why it should be thought desirable to insist on a hurried decision in order to maintain our present system, not of Free Trade, but of Free Imports. (Cheers.) In conclusion he expressed the hope that before long there would be more direct representation of medical men in Parliament.

Mr. Eugene Wason, M.P., who replied, thanked Sir Henry for his interesting and lucid speech. He had been a member of the Committee on National Expenditure, and by their report to be issued next week they would see that many of Sir Henry's suggestions had been anticipated. As to the question of Free Trade, he was a "convinced Free Trader," but then so much depended on the definition of the term. He hoped, however, that all discussions on the subject would be conducted with fairness and good humour on both sides.

Mr. P. J. Freyer proposed "The Association of British Postal Medical Officers," to which the Chairman briefly responded, thanking his confrères for the honour they had that day conferred on him by selecting him for the fourth time for the office of President.

The toast of "The Guests" was proposed by Dr. Sheldon and responded to by Sir Gilzean Read, after which the proceedings terminated.

